# 3D Trajectory Planning Method for UAVs Swarm in Building Emergencies

**DOI:** 10.3390/s20030642

**Published:** 2020-01-23

**Authors:** Ángel Madridano, Abdulla Al-Kaff, David Martín, Arturo de la Escalera

**Affiliations:** Intelligent Systems Lab (LSI), Universidad Carlos III de Madrid, Avnd. Universidad 30, 28911 Leganés, Madrid, Spain; amadrida@ing.uc3m.es (Á.M.); akaff@ing.uc3m.es (A.A.-K.); dmgomez@ing.uc3m.es (D.M.)

**Keywords:** UAV, 3D probabilistic roadmaps, trajectory planning, multi-robot systems

## Abstract

The development in Multi-Robot Systems (MRS) has become one of the most exploited fields of research in robotics in recent years. This is due to the robustness and versatility they present to effectively undertake a set of tasks autonomously. One of the essential elements for several vehicles, in this case, Unmanned Aerial Vehicles (UAVs), to perform tasks autonomously and cooperatively is trajectory planning, which is necessary to guarantee the safe and collision-free movement of the different vehicles. This document includes the planning of multiple trajectories for a swarm of UAVs based on 3D Probabilistic Roadmaps (PRM). This swarm is capable of reaching different locations of interest in different cases (labeled and unlabeled), supporting of an Emergency Response Team (ERT) in emergencies in urban environments. In addition, an architecture based on Robot Operating System (ROS) is presented to allow the simulation and integration of the methods developed in a UAV swarm. This architecture allows the communications with the MavLink protocol and control via the Pixhawk autopilot, for a quick and easy implementation in real UAVs. The proposed method was validated by experiments simulating building emergences. Finally, the obtained results show that methods based on probability roadmaps create effective solutions in terms of calculation time in the case of scalable systems in different situations along with their integration into a versatile framework such as ROS.

## 1. Introduction

The high versatility and applicability of mobile robots, both industrial and military, has led to an increase in their use over the last decade. In addition, the advances of the electronics technologies enhanced the intelligence and consequently raised the level of these vehicles; improved the energy sources, such as LiPo batteries; and reduced the weight and cost of electronic components, sensors, and actuators.

Unmanned Aerial Vehicles (UAVs) and Unmanned Ground Vehicles (UGVs) have positioned themselves at the forefront of important fields of research oriented to various tasks, such as infrastructure inspection [1,2,3,4], surveillance [5,6], search and rescue [7,8,9], or delivery [10,11].

Although both vehicles have similarities in terms of their ability to move autonomously or be remotely controlled, they have different characteristics in terms of load capacity, speed, accessibility, or maneuverability. These differences introduce the necessity of using Heterogeneous Multi-Robot Systems (MRS), formed by these types of vehicles in order to act in a wide variety of tasks.

For autonomous MRS able to carry out a series of works in a specific scenario, it is essential to establish trajectories that allow the safe movement in the environment, avoiding both static and dynamic obstacles. For this point, trajectory planning becomes a key aspect for this type of systems to be able to undertake more complex missions in shorter periods [12].

The planning and the set of actions that allow multiple vehicles to achieve an objective can be divided into two phases.

- Task Planning: For a set of vehicles and set of destinations, the most optimal vehicle-objective combination is established, considering aspects such as the speed and endurance of the vehicle, the distance to the goal, and the task to be undertaken [13].
- Trajectory Planning (or required movements): Once the objective has been assigned, safe and efficient paths are generated for one or more vehicles to be able capable of reaching this location [14].

Focusing on the second phase, the need arises to develop methods that allow planning of the trajectories with MRS, considering possible collisions with both static and dynamic obstacles.

This work presents an extension of the work “Multi-Path Planning Method for UAVs Swarm Purposes” [15], which includes a trajectory planning method for a swarm of UAVs operating in a structured environment. Although the generated paths are considered 3D, the exploration of the environment is carried out in 2D using a plan view image of the environment. Once the path is generated, in which all the obstacles present in the environment are considered to be of fixed height, a different and constant height is assigned to each UAV.

The present work focuses on the exploration and establishment of trajectories considering an environment in 3D, from the mapping and generation of the occupation map of the environment. In this way, and through the development of algorithms based on Probabilistic Roadmaps (PRM), a solution is provided for the safe autonomous movement of the different vehicles. The main idea is to design an architecture that allows a swarm of UAV to act efficiently and safely within a complex environment, such as supporting ERT in rescue work or urban emergencies.

This work is structured as follows. Section 2 contains a review of recent work in the literature related to path planning for autonomous vehicles working individually or cooperatively (Section 2.1) and the use of UAVs in emergency work (Section 2.2). Section 3 focuses on describing the proposed 3D trajectory planning method, together with the architecture developed to integrate the results into the drone fleet for future simulations and real tests. Section 4 analyzes the results obtained, showing how this type of algorithm allows a rapid response in the case of emergency for a scalable team of autonomous vehicles. Finally, Section 5 contains conclusions and details of future developments.

## 2. Related Works

### 2.1. 3D Path Planning

Different studies have been conducted to solve the problem of planning multiple trajectories for multiple vehicles to achieve different objectives. These methods included those based on graphs or potential fields, probabilistic methods, or deep learning. Some of these methods allow exploring large areas of interest and reducing the computational time, such as Probabilistic Roadmaps (PRM). This work focuses on the PRM algorithm, due to its advantages compared with other algorithms (e.g., A*, Rapidly-Exploring Random Tree (RRT), bidirectional RRT (bRRT), and Genetic Algorithms (GA)) in terms of both shortest time and distance parameters [16].

Reviewing the recent state of the art in 3D trajectory planning for MRS, it is found that probabilistic methods continue to adopt effective solutions to this problem. These methods are characterized by building a graph from the connection of randomly generated nodes in the workspace free of obstacles. In this way, a graph of a certain number of nodes is created, which makes it possible to find a path that connects a starting point with an endpoint. Among the advantages of this type of methods is the possibility of quickly exploring a large environment and reusing the mesh created to find multiple connections within the explored area. On the contrary, it presents the problem of not being complete, i.e. there is the possibility of not finding a solution even if it exists, given that random exploration can leave transit areas undiscovered, as is the case of narrow corridors. Besides, in the case of PRMs, there is the talk of a passive method, i.e. it presents the need to use a second algorithm that finds, among all possible paths, the optimal between the initial configuration and the desired one [17].

DIAS et al. [18] established a behavior between two PRM-based algorithms for the planning of UAV trajectories in search and rescue environments. They implemented PRM algorithms in real-time that can be applied in real scenarios, although for the moment the results are collected in the Modular Open Robots Simulation Engine (MORSE) simulation environment.

Among the recent works related to the use of PRM for path planning is [19]. Although it is not oriented to UAVs, it presents an algorithm based on PRM to establish optimal paths in radioactive environments in order to minimize the exposure of people in hostile environments of this type. Similarly, Upadhyay et al. [20] used a PRM in 2D and then algorithm A* to find an optimal set of paths for a system formed by multiple robots, which are coordinated with a UAV.

Focusing the research on those works related to the 3D planning trajectories is [21], in which the information is taken from a 3D occupancy map to, as in the present work, generate a 3D PRM that allows finding collision-free paths. In our case, a similar approach is followed but oriented to a multi-UAV system. Following this line of research, Samaniego et al. [22] used hybrid or PRM improvement methods to plan 3D routes for UAVs.

As indicated above, there are methods based on a wide variety of implementations that allow a solution to be found to the problem of 3D trajectory planning. Among the methods based on graphs, Guastella et al. [23] combined a 3D grid and the A* algorithm to obtain optimal routes for the accomplishment of missions on the part of a UAVs swarm. In addition to this algorithm, a negotiation phase is included to find the combination of trajectories that minimizes the total length of all the paths.

As for the methods based on potential fields, Falomir et al. [24] presented a 3D mobility model for autonomous swarms of collaborative UAVs; they also included a global 3D planner, all validated in OMNeT++ with simulations in urban environments.

### 2.2. Emergency Drones Applications

In addition to reviewing the recent works in the field of 3D path planning, it is necessary to know the current status of the use of UAVs in emergency, either individually or cooperatively and, specifically, in those tasks performed in urban areas or buildings, such as the one used in presented in this work.

Focusing on the use of UAVs in fire-related emergencies, most of the works present techniques such as the one in [25], in which on board cameras are mounted on UAV, to detect smoke and track emergency bodies. Generally, works related to fires are oriented to the use of aerial vehicles as carriers of cameras and sensors to detect or monitor elements related to the fire, such as smoke columns, flame fronts, vegetation near a fire, and emergency equipment in the area [26,27,28].

Concerning emergencies and UAV applications in urban environments, several works have been introduced. The work presented in [29] covers the use of the UAVs to transmit live videos to emergency services in traffic accidents. Another applicability of UAVs in urban environments, in both emergency and non-emergency situations, is the possibility of establishing air communications networks, to improve or re-establish telecommunications links [30]. Finally, for example, Rokhsaritalemi et al. [31] established planning of trajectories in drones based on geographic information to carry out 3D models of urban environments.

In line with what is included in this section, this work presents the planning of 3D trajectories for a system made up of multiple UAVs in structured environments and urban environments. Specifically, it includes a case study in which a fleet of drones, controlled with the Pixhawk autopilot [32], is capable of achieving different objectives or emergencies within a building of different floors. Besides, the implemented method has been established to achieve scalability and interoperability of the equipment.

## 3. 3D Multi-Trajectory Planning Based on PRM

The idea of this work is to present a set of algorithms that allow, from 3D occupation maps, to generate a set of optimal trajectories so that, autonomously, different drones can reach different locations of interest within the work area. To do this, as detailed in this section, algorithms based on PRM and Octomap [33] are used together for the exploration of a 3D environment and to establish the optimal set of routes for each aircraft to reach a specific objective in such a way that the mission as a whole is developed in the most efficient way possible.

In addition to the extension of the work from 2D to 3D, the integration of all methods in Gazebo’s simulation environment is included, allowing the visualization and testing of the algorithms before the implementation with real UAVs. Although the integration into Gazebo was already part of the previous work, in this new proposal, the Pixhawk software in the loop is used instead of hector_quadrotor package [34].

**Figure 1:**
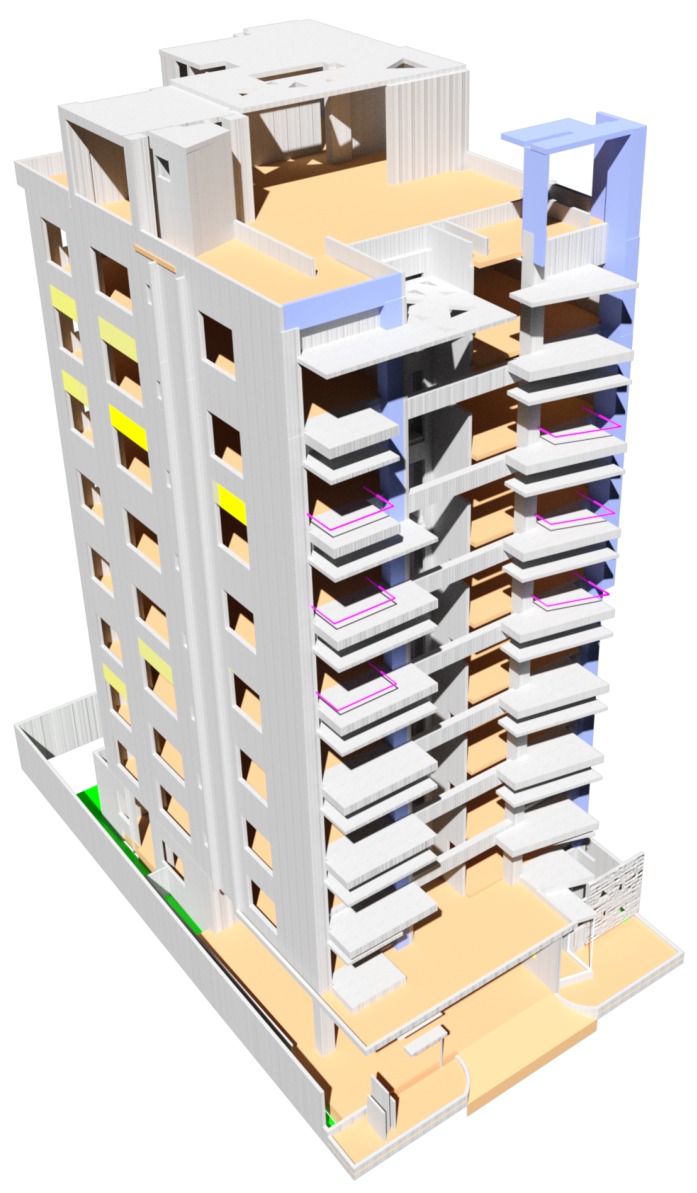
3D model of the building for simulation.

Planning safe and efficient trajectories is a crucial step for a MRS to complete a set of tasks. In this case, the paths generated are oriented to the use of swarm of UAVs to support ERT in urban areas, especially in cases of large buildings with a large area to cover (Figure 1). Thus, a centralized and scalable architecture is presented, in which, using the Robot Operating System Framework (ROS) [35] and the MavLink communication protocol (MAVROS), a heterogeneous swarm of UAVs is established that can reach, in a short interval of time through safe and efficient routes, strategic places for the support and collection of information necessary to increase the response and success of the tasks of the ERT.

To achieve this objective, it is necessary to complete a set of phases, considering two cases: labeled case, in which each UAV knows what target it should go to; and unlabeled case, in which the UAV-trajectory set is established to minimize the total distance travelled by the swarm throughout the mission.

### 3.1. 3D Occupancy Map Generation

The first step to achieve path planning is to have a 3D occupancy map of the work area, where the swarm must operate. For this, the information obtained from Octree algorithms is used. Octree is a hierarchical data structure that subdivides the space into cubes or voxels until a certain resolution is reached. One of the properties of these volumes is to know if it is free or occupied and, using this information, the exploration of the work area is carried out.

For the creation of the occupation map of the environment, the Binvox library [36,37] and the Octomap library [33] are used. Thus, from the 3D model of the environment, an Octree or occupation map is obtained to generate the PRM of the possible paths to reach the desired locations.

**Figure 2:**
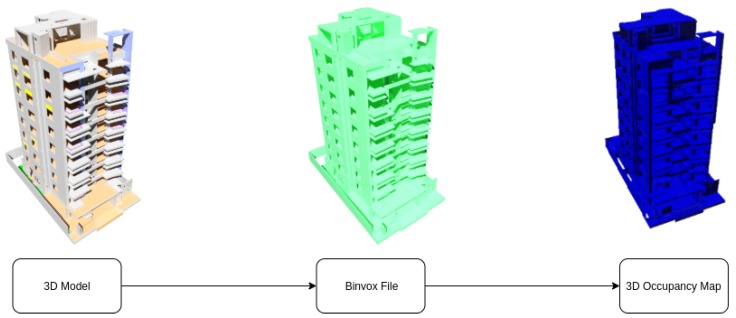
Obtaining 3D occupancy diagram.

Thus, as shown in Figure 2, the process followed to obtain the Octree involves converting the 3D model to a binvox file. This file is a binary voxelization of the object in 3D that contains information about which zones are occupied and which are free. Then, through the Octomap library, an Octree is generated to be used format to act as an input in the exploration of the environment.

Another possibility for obtaining the occupation map is to deploy throughout the work area a set of vehicles equipped with sensors, in order to obtain a complete mapping, for which it would be necessary to establish full coverage and sensorial fusion algorithms that compile the dataset from each of the vehicles and generate a complete map.

### 3.2. Exploration of the 3D Environment Based on PRM

Once the 3D occupancy map is obtained, a probabilistic algorithm is implemented to explore the area and, provided a set of possible solutions, to obtain the shortest paths. In this work, the A* [38] algorithm is used.

As mentioned above, the idea is to establish an algorithm based on PRM to explore the environment. This method aims to reduce the computational time of exploration to be done once. This is independent to the number of vehicles used. In other words, the exploration (computational expensive) is performed one time, and then A* algorithm finds the shortest paths for each vehicle based on exploration. For this reason, this type of algorithms is considered a good solution for the problem of trajectory planning in MRS, and, similar to other probabilistic methods, is considered for scenarios of great dimensions.

As shown in the Results Section 4, it is important to create a number of nodes to keep a balance between the computation time and the extension of the area to be covered. However, it is worth mentioning that it is possible not to find the solution, due to the incomplete sampling of the area.

**Figure 3:**
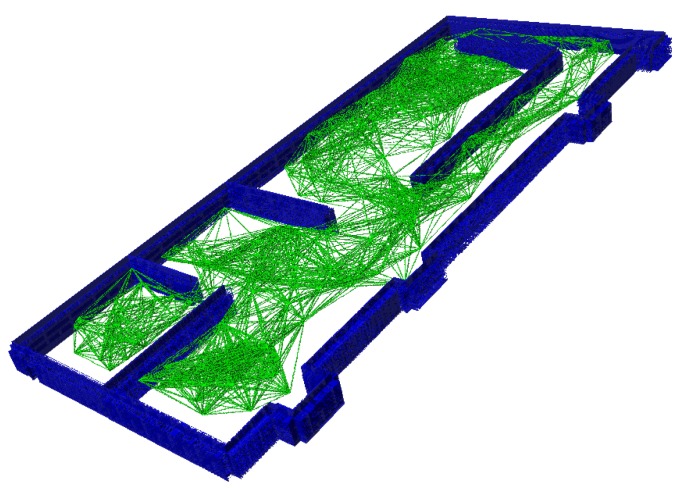
3D Probabilistic Roadmaps.

The steps followed for the exploration of the 3D environment are shown in Algorithm 1 and detailed below.
**Algorithm 1:** 3D probabilistic roadmaps.**Input**: 3D Occupancy Map map **Output**: 3D Probabilistic Roadmap (Figure 3). 
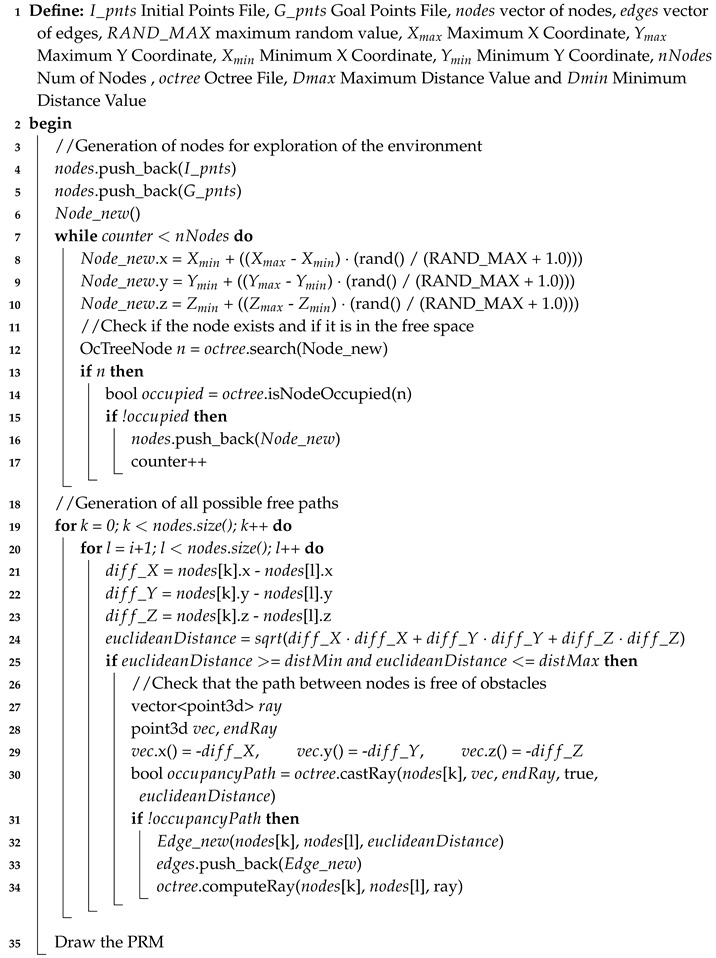


The first step is to define a set of variables required by the algorithm:

- I_pnts: A file containing the initial positions of the vehicles. In this case, these positions are established concerning (0,0,0) of Gazebo. In the real application, the conversion of the global coordinates to a local reference system is required taking into account the initial position of one of the vehicles. Knowing the position of the goals and each of the vehicles, it is possible to establish a common reference system for all agents involved in the mission.
- G_pnts: A file containing the final positions of the goals to be achieved. As in the previous case, these coordinates appear in meters with respect to the coordinates of the origin simulation. As indicated, the idea is to introduce intermediate processing that is in charge of taking the position of each vehicle and the locations to reach, referencing all data to a common reference system.
- Xmax, Xmin, Ymax, Ymin, Zmax, Zmin: Sets of the maximum and minimum values of the coordinate of a node in the *X*, *Y*, and *Z* axes, respectively. These values are fixed taking into account the size of the Octree, and they are extracted through functions collected in the Octomap library.
- Nnodes: Variable that allows parameterizing the number of nodes to explore the environment. As mentioned above, it is important to establish a balance between computing time and a high possibility of finding all possible solutions. The greater is the number of nodes, the longer is the computation time, but the greater are the chances of finding a solution and making it as short as possible. If the number of nodes is decreased, the computation time is minimized, but the possibility of finding a possible solution is reduced.
- Dmax, Dmin: To improve the creation of the PRM, these values are introduced in such a way that the generated edges are in a range of distance. That leads to eliminate the path of two very close nodes. The maximum distance is limited to increase the possibility to find a free collision path. In the case of the number of nodes, it is necessary to establish a balance between these distances and the total size of the Octree.

Once the algorithm has been parameterized, a set of phases is established within the method that allows generating a finite set of possible paths.

1. Generation of nodes: Nodes are generated with random coordinate values, as shown on Lines 8–10 of Algorithm 1. To do this, it is necessary to use the variable RAND_MAX, which corresponds to a constant of C++ that returns the maximum value of the function rand(). Once the node is generated, the next step is to look for this node inside the Octree, and check if it corresponds to free space. If so, it is added to the set of PRM nodes.
2. Edge generation: The next step is to establish all possible connections between the generated nodes. To achieve this, first the algorithm checks if the nodes are at a valid distance according to the established range, and then verifies if the line connects both nodes is free collision path. This process is carried out using the Octomap libraries, which have functions that allow throwing rays from one point to another, receiving if the ray is hitting an obstacle or not, and checking that the end of the ray is the destination and not an intermediate point. In addition, this function takes into account the direction in which the beam must be launched and its distance, thus it ensures that the path sought is between the two nodes generated. Once the check is done, the edge is saved.

**Figure 4:**
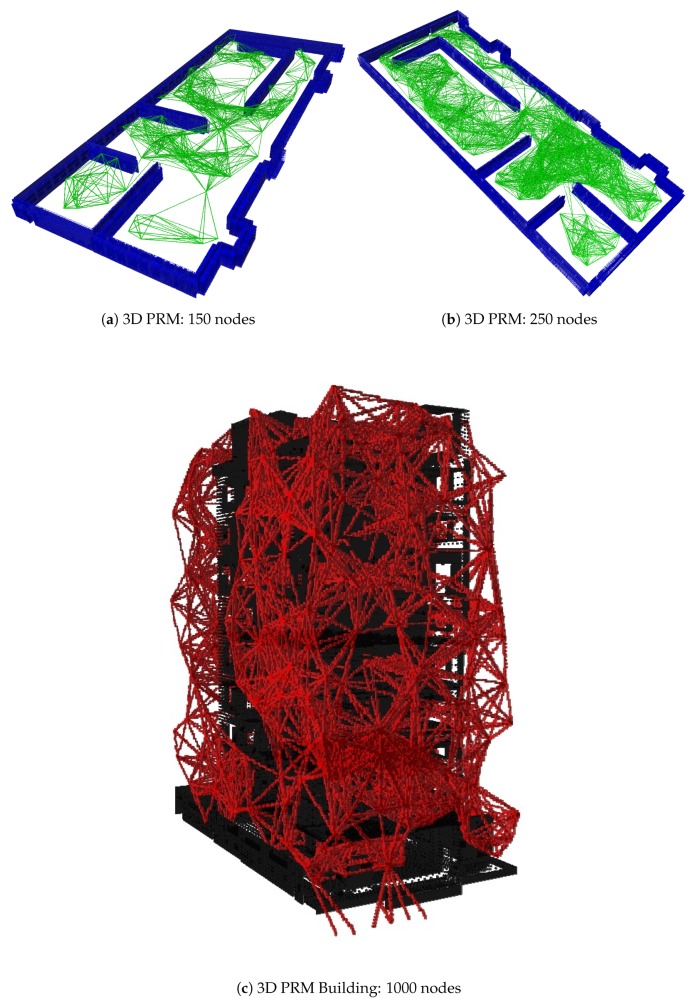
3D PRM output.

Figure 4 shows several examples of the output of the 3D PRM generation algorithm for two different cases. Figure 4a,b shows the graph generated for a structured environment in the case of generating 150 and 250 nodes, where the obstacles are represented in blue and the possible paths are in green. Figure 4c shows an example of the output of the case study of emergencies in buildings with obstacles in black and possible trajectories in red, where 1000 nodes generated a finite number of solutions throughout the building.

### 3.3. Generation of Paths

The last step is to establish the optimal path to join the initial position of the UAVs to the goals.

As stated in the previous work, this last step is oriented to different situations, and the results are focused on the labeled and unlabeled cases.

Although there are different situations, the generation of paths has a common development for all cases, and adds a procedure in the case of planning trajectories for unlabeled cases. The common part consists in the use of the algorithm A* [38] to find the optimal path once the PRM is generated. The reason for this second phase is the passive character of the PRM algorithm, which randomly explores the environment but is not able to provide a solution.

#### 3.3.1. Generation of Paths for the Labeled Case

A labeled case is the assignation of a number of vehicles to accomplish a mission or a set of tasks. In this case, it is required to find and assign the optimal trajectory between start and goal points of the mission.

In a real case (i.e., urban emergencies), a heterogeneous type of vehicles with different characteristics and sensors is usually used. Thus, some vehicles are more suitable than others for a specific task. Therefore, it is required to have a prior algorithm for weighing all these conditioning factors and establishing which vehicle is the best to cover a certain task. This algorithm is known as Multi-Robot Task Allocation (MRTA), and it is responsible for assigning the optimal robot–task combination, considering aspects such as the speed of the vehicle, the urgency of the mission, the endurance of the vehicle, the load capacity, or the type of information that can be captured with the onboard sensors. Ideally, task assignment methods should be combined with path planning methods that first allow robot–task pairs to be fixed and then find the most efficient way to achieve them. This work has focused only on the problem of generating and seeking for the optimal path, independently of the Task Allocation problem. In this paper, this search for the optimal path is done using the A-Star algorithm for each drone–target pairing, reusing the created network in each case.

#### 3.3.2. Generation of Paths for the Unlabeled Case

The unlabeled case is the most complex case studied in this paper. It has a set of *N* UAVs to undertake a number of missions (*N*), but, unlike the previous case, there is no prior allocation. Therefore, additional methods must be introduced to establish an optimal allocation between the different tasks and vehicles. In this case, this work focuses on methods that provide a solution for each vehicle, considering the total distance traveled by the system as the key aspect to minimize.

**Figure 5:**
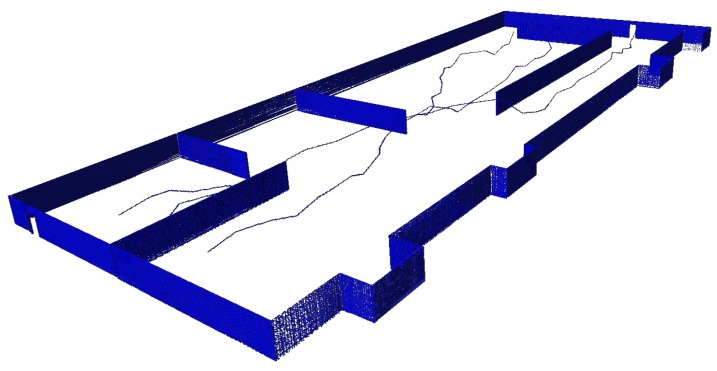
Solution for multiple tasks and multiple vehicles.

Therefore, it is necessary to look for a method that allows making an assignation between two different categories, minimizing a common factor. To deal with this problem, the Hungarian method [39,40] is used to minimize allocation problems. Then, for the unlabeled case, an intermediate phase is introduced to carry out the assignment by implementing the Hungarian method for minimizing the total distance traveled by the MRS. Therefore, once the Hungarian method is applied, a task assignment is produced and the optimal set of paths is obtained for each vehicle to achieve its goal (Figure 5).

**Figure 6:**
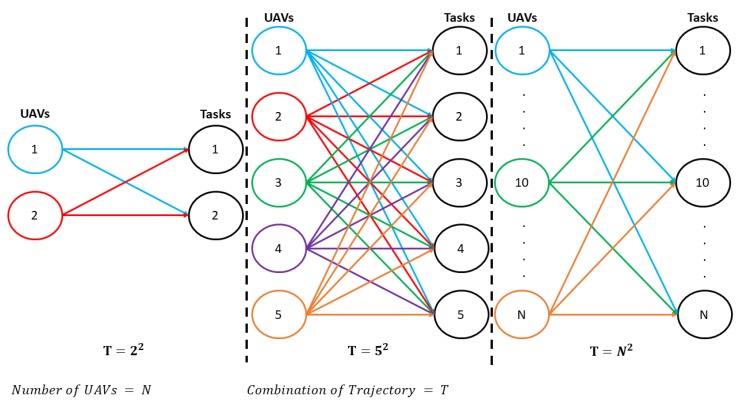
Combination of trajectories to be optimized with the Hungarian Method.

It is necessary to consider that, when *N* increases, the complexity of the problem to be solved increases exponentially, as shown in Figure 6. The figure shows that, for two UAVs assigned to undertake two tasks, the Hungarian method must process a 2×2 matrix with four possible solutions; however, in the case of five UAVs, there are 25 possible paths. Note that the Hungarian method works with $N^{2}$ solutions.

### 3.4. Collision Avoidance

Once the optimal combination of paths is obtained, it is necessary to ensure that the UAVs move along these trajectories without colliding with each other.

A method is developed to check the position and modify the speed of each UAV, if two or more of them approach the same point. In this way, the UAV moves autonomously at a speed set by the autopilot, until two or more UAVs approach each other below a safety margin set by the user. In this case, a reduction in the speed of one of the two UAVs is applied until the distance between them is safe. In the case of more than two UAVs, different speed reductions are set for each UAV except one (Algorithm 2).

In this work, the ID of each vehicle should be considered to give a priority to the one with the highest ID, reducing the speed of the lower ID (Algorithm 2, Line 16).
**Algorithm 2:** Collision avoidance between UAVs.
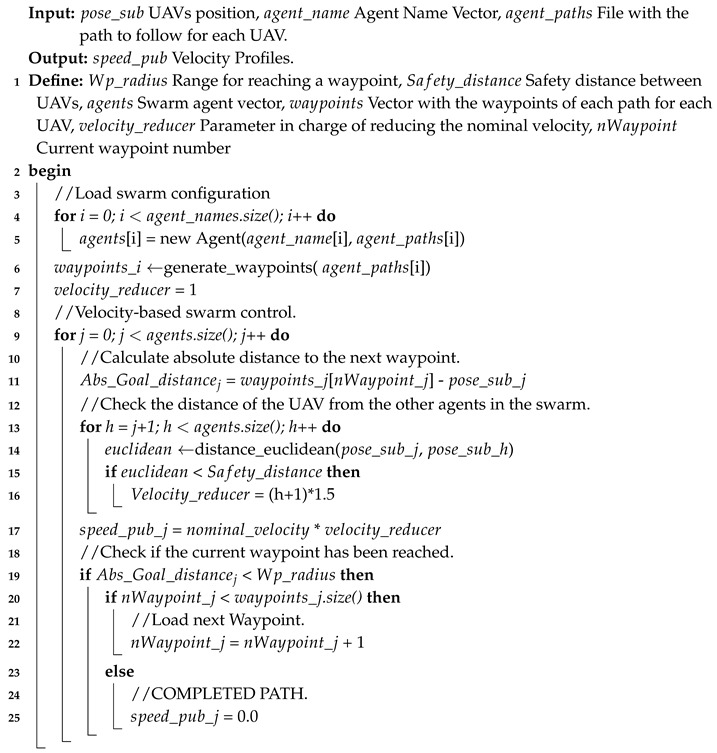


## 4. Simulation and Results

To validate the proposed algorithm, several simulations were performed. This section details the simulation environment, architecture, and discusses the obtained results.

### 4.1. Control Architecture and Simulation

In the field of autonomous vehicles, especially UAVs, simulations are crucial to test and validate the implemented algorithms because any minimal error in the methods developed usually leads to a failure in the control of the UAV, and may lose sensors or elements of high economic value. For this reason, a software architecture is designed in Gazebo’s simulation to allow testing the different MRS missions. In the area of robotics and, more specifically, in that related to UAVs, simulation becomes a key aspect as a previous step to the implementation of an algorithm in real systems because any minimal error or failure of the methods developed in mid-flight usually leads to the loss of control of the system. If, in addition, the idea is to put into operation UAVs in which the payload is sensors or elements of high economic value, the simulation becomes a crucial element to ensure the proper functioning of all systems. For this reason, a software architecture was generated through Gazebo’s simulation environment that allows simulating the different MRS missions looking for the greatest similarity to a real test.

To this end, using as a framework ROS, a centralized architecture was established, in which a single computer takes the control and exchange of the information with each of the UAVs that build the MRS. ROS is usually used due to its capability to implement and develop collaborative robotic software to establish a scalable and robust information communication network. In addition, ROS has important developments in the acquisition of information from sensors, or communication protocols such as MAVROS, which allows the communication with the Pixhawk autopilot via MavLink protocol.

**Figure 7:**
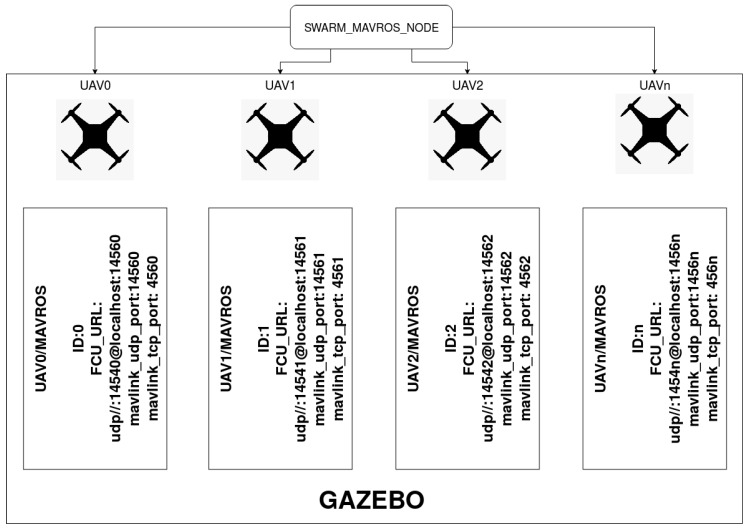
Control architecture diagram.

As shown in Figure 7, a central node was created to provide a position control of the UAVs via MAVROS, which allows the simulation of PX4 firmware, the development of the mission, and the visualization in Gazebo.

The input of this node is a list of names of all the UAVs, the initial position of each one, and the file that contains the paths of the UAVs.

Once these parameters are loaded, the node changes the state of the UAVs to Offboard mode, arms their motors, and sends the waypoints to follow. Moreover, this node subscribes to the GPS position of each UAV to check if the waypoint has been reached or not. Finally, once the trajectory has been completed, a landing maneuver is performed.

The node is configured to be scalable to add or remove UAVs of the MRS. This is achieved by the config file that collects the name of the UAVs, a waypoint file, and the initial position of the UAV. Once the local and global reference systems are added, it is not necessary to include the initial position in the configuration file, and it will only be necessary for the creation or elimination of UAVs to establish a name and the path of the file that contains the waypoints of the trajectory.

### 4.2. Results

**Figure 8:**
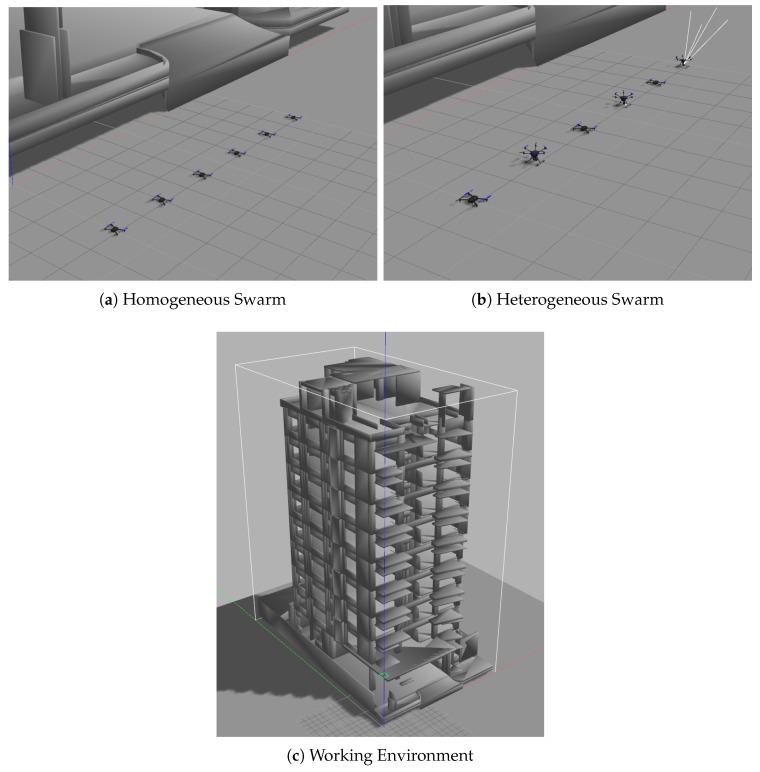
Simulation environment.

As shown in Figure 8, the working area simulated a nine-floor building with multiple rooms on each floor. This building has the dimensions of (49, 24, 41) m for (height, width, length), which provides 48,216 m${}^{3}$ of working area.

It is important to emphasize that the computational time illustrated in the figures is the average of 10 repetitions for the same case. In this way, more reliable results are compared, especially in the unlabeled case, in which the computational time of the Hungarian method is affected by the total number of the solutions.

**Figure 9:**
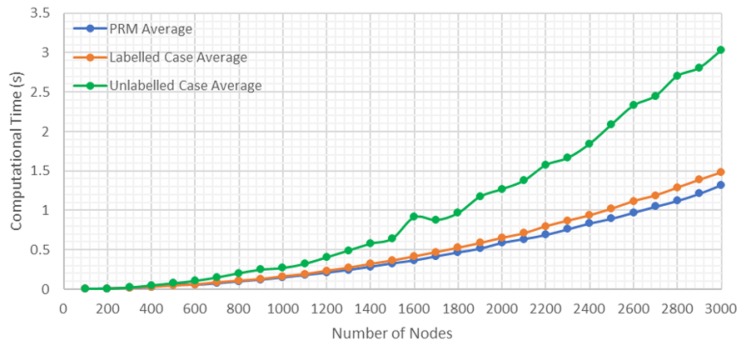
Results of the computation time according to the number of nodes.

The first results are shown in Figure 9, in which the average computational time is recorded according to the number of nodes used to explore the environment. In the figure, three case studies are included:

- **PRM Average:** It is the average computational time for generating PRM without considering if it is labeled or unlabeled case.
- **Labeled Case Average:** It is the average computational time for generating the solution for the labeled case.
- **Unlabeled Case Average:** It is the average computational time for generating the solution for the unlabeled case.

**Figure 10:**
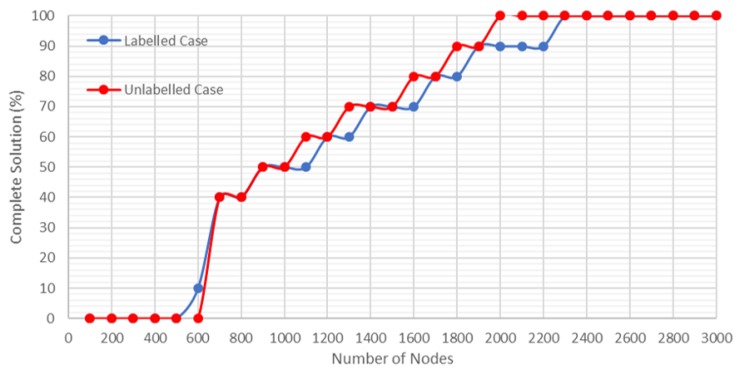
Percentage of times a solution is found as opposed to the number of nodes employed.

In this study, all the results were obtained by carrying out a number of tasks equal to 10. In Figure 9, the greater is the number of nodes used, the greater is the time required to carry out the exploration, and the greater is the probability of finding a complete solution (Figure 10).

In addition, it is observed that the computational time of finding the optimal path by A* is low. This is because the time used to generate the PRM and the total time used in the labeled case are similar, and there is a slight difference when the number of nodes is higher than 2500 nodes. On the other hand, for the unlabeled case, it is shown how the Hungarian method increases the computational time, although it finds a solution for a smaller number of nodes (Figure 10).

As shown in Figure 9 and Figure 10, for 2300 nodes, in the case of an emergency for a building with nine floors and a volume of 48.3 km${}^{3}$, the time required to find a solution for any of the situations studied in the labeled case is around 0.7 s, and in the unlabeled case is slightly more than 1 s.

**Figure 11:**
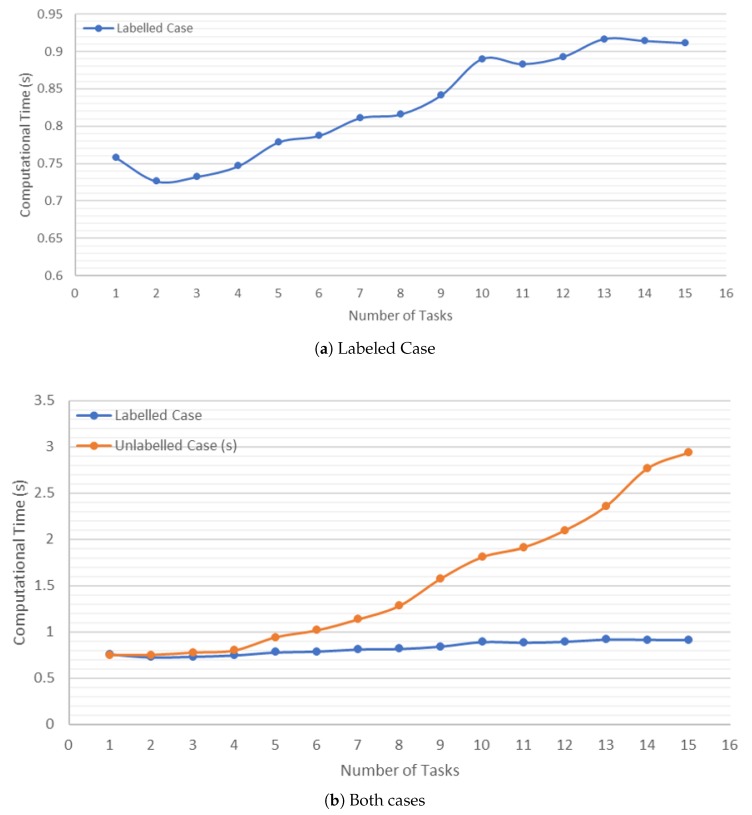
Computation time depending on the number of tasks and agents involved in the mission.

In Figure 11a, it is noted that, in the labeled case, the increase in tasks does not cause increment in computational time. This shows that the implemented algorithm is scalable. When the number of tasks increased from 1 to 15, the computational time used to find a complete solution increased from 0.7 to 0.9 s.

The scalability is achieved by exploring the environment once, and then reusing the generated PRM for each UAV finding the optimal path.

Figure 11b shows how, in the unlabeled case, the computational time increases quadratically with respect to the increment in the number of tasks, due to the Hungarian method. Although there is an increment in time, the result from Hungarian method can be considered sufficiently good, because the possible solutions increase exponentially while the computational time increases in a quadratic way. This increment in computation time helps to minimize the total distance traveled by the MRS.

To complete the analysis and comparison between the two cases, it is important to know how the paths increase. For this reason, it has been decided to include in this section a comparison between the total distance of all the trajectories calculated for both cases and on the same PRM depending on the number of tasks and aircraft participating in the mission. In addition, to complete the paper, whether the distance travelled changes due to an increase in the number of nodes used for exploration is considered.

**Figure 12:**
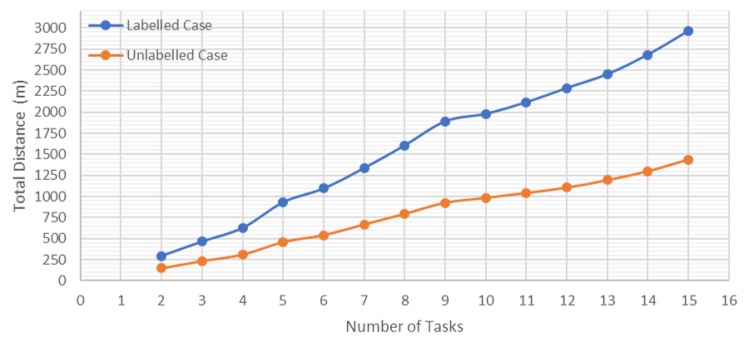
Total travelled distance vs. number of tasks.

Figure 12 shows a comparison of the total distance travelled by the UAVs swarm of the same PRM according to the number of tasks. It can be seen how the application of the Hungarian method with the unlabeled case minimizes the total distance travelled, decreasing the difference between the two cases, and providing more efficiency when the number of tasks increases.

**Figure 13:**
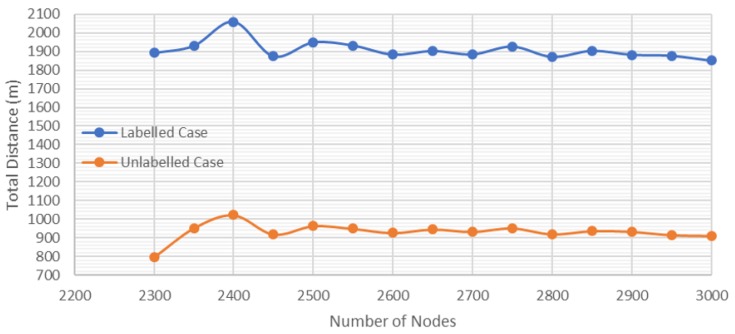
Total travelled distance vs. number of nodes.

Figure 13 shows an example of the variation of the total distance with respect to the number of nodes for nine tasks. When considering from 2300 to 3000 nodes, it is almost fixed. Thus, it is not necessary to increase the number of nodes to minimize the distance.

Thus, working with the labeled case using MRTA algorithm is possibly a better option. While this increases the response time, the allocation is more efficient, when considering other factors, such as payload capacity, endurance, speed, and emergency level, to establish a UAV–task combination.

Another factor that can be set is the size of the UAV. To achieve this, a parameter that allows generating a safety margin around the obstacles is included. Thus, the possible waypoints of the paths are located in collision-free space for a UAV. Therefore, with the application of the safety margin, if one UAV accomplishes its task, any UAV of the same size can accomplish the same task. When emergencies occur inside the building, when the size of the UAV increases, the reachability decreases.

Table 1 presents a set of 10 tasks and whether they are reachable based on the UAV size. This characteristic opens up a range of possibilities for heterogeneous MRS, since it provides information about the maximum size of the UAV with which to undertake each of the tasks. The results gathered in the Table 1 indicate “Yes” in the case that at least one of the UAVs is capable of reaching the task and “No” if none of the UAVs with that size can reach the objective.

**Figure 14:**
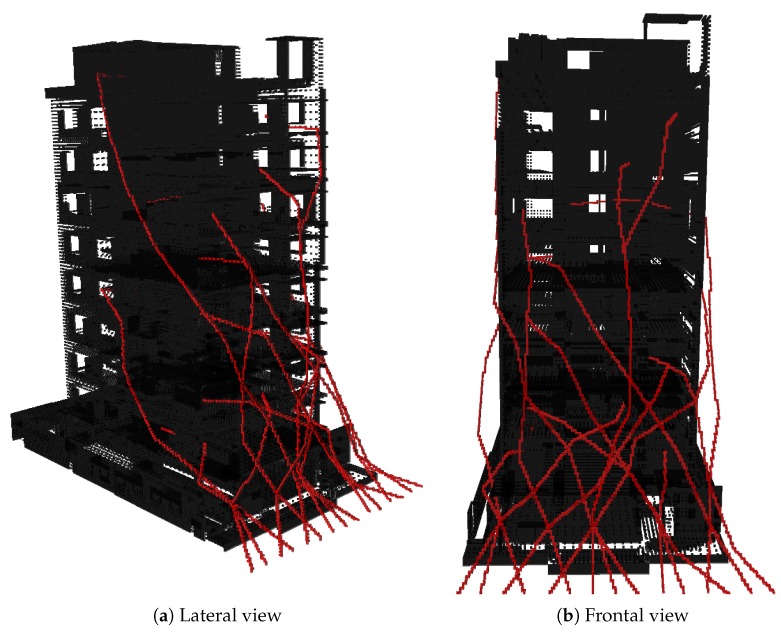
Set of paths for a swarm of 10 vehicles.

**Figure 15:**
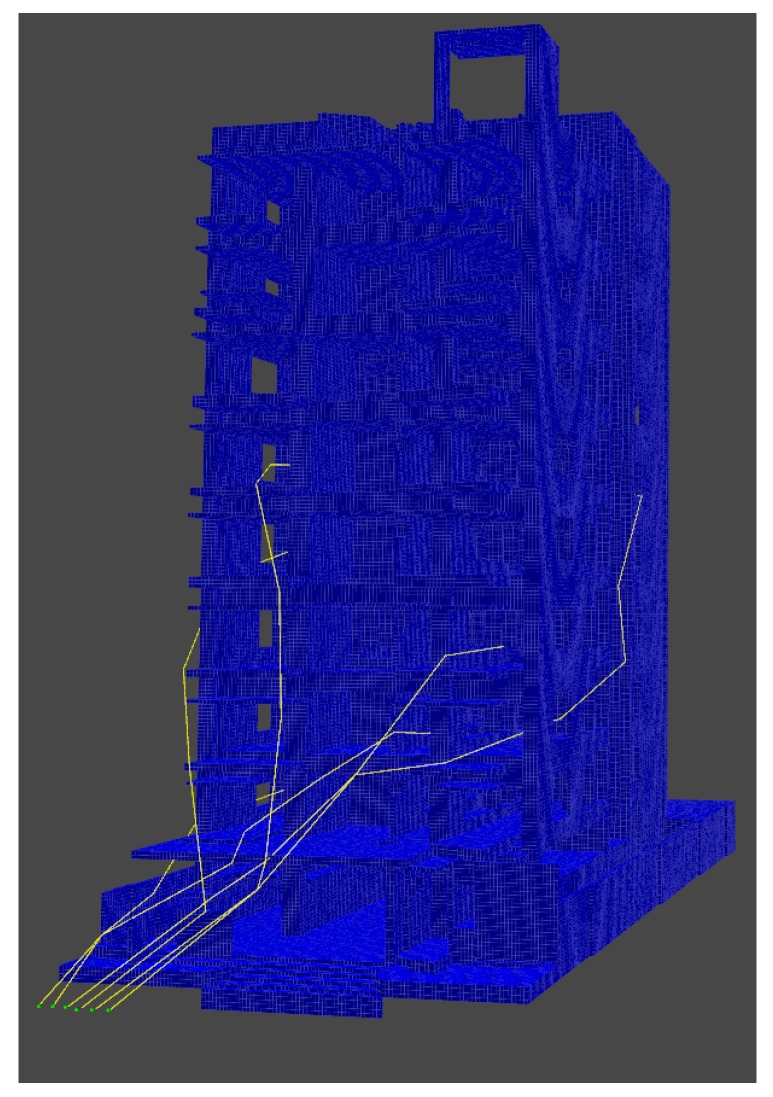
Visualization of the complete mission in RVIZ.

Figure 14 shows the Octree of the whole building, and the set of trajectories followed by each of the UAVs, from its start points to the goals location inside the building. Figure 15 shows the point-cloud of the building.

## 5. Conclusions

This work presents a method of 3D trajectory planning for a UAV swarm based on PRM for urban emergencies applied on labeled and unlabeled cases, supporting an Emergency Response Team. In addition, this work includes ROS architecture for simulation, in which a communication bridge between ROS and MavLink protocol is established.

The obtained results from simulation demonstrate the scalability of applying this method on heterogeneous MRS, which has the ability to generate a set of optimal solutions in a short time. The proposed algorithm details the creation of a single PRM and its reuse for each of the UAV, allowing to reduce the increment of computational time. In addition, the application of the Hungarian method for the tasks allocation allows minimizing the total distance travelled by UAVs.

Future research will focus on the development of a node responsible for referencing the UAVs’ initial and task positions to global system, instead of Gazebo (0, 0, 0) origin. Moreover, the MRTA algorithm for labeled case will be developed for efficient allocation.

Another future research will be to mount onboard sensors to detect the dynamic obstacles in real time and modify the preset path if necessary. In addition, the proposed algorithms will be implemented in complete coverage area and swarm formation problems.

## Figures and Tables

**Table 1:** Reachability of the tasks considering the size of the UAV.

Safety Margin as UAV Size (m)	T1	T2	T3	T4	T5	T6	T7	T8	T9	T10
**0.5**	Y	Y	Y	Y	Y	Y	Y	Y	Y	Y
**1.0**	Y	Y	Y	Y	Y	Y	Y	Y	Y	Y
**1.5**	Y	Y	Y	Y	Y	Y	Y	Y	Y	Y
**2.0**	Y	Y	Y	N	Y	Y	Y	Y	Y	Y
**2.5**	Y	Y	Y	N	Y	Y	Y	N	Y	Y
**3.0**	Y	Y	N	N	Y	Y	Y	N	Y	Y
**3.5**	N	Y	N	N	Y	N	N	N	Y	N
**4.0**	N	N	N	N	Y	N	N	N	N	N
**4.5**	N	N	N	N	Y	N	N	N	N	N
**5.0**	N	N	N	N	N	N	N	N	N	N

T = Task, Y = Yes, N = No.
